# Effective Engagement of Adolescent Asthma Patients With Mobile Health–Supporting Medication Adherence

**DOI:** 10.2196/12411

**Published:** 2019-03-27

**Authors:** Richelle C Kosse, Marcel L Bouvy, Svetlana V Belitser, Tjalling W de Vries, Piet S van der Wal, Ellen S Koster

**Affiliations:** 1 Division of Pharmacoepidemiology and Clinical Pharmacology Utrecht Institute for Pharmaceutical Sciences Utrecht University Utrecht Netherlands; 2 Department of Pediatrics Medical Centre Leeuwarden Leeuwarden Netherlands; 3 Umenz Benelux BV Hilversum Netherlands

**Keywords:** adolescent, asthma, medication adherence, pharmacists, telemedicine

## Abstract

**Background:**

Mobile health (mHealth) apps have the potential to support patients’ medication use and are therefore increasingly used. Apps with broad functionality are suggested to be more effective; however, not much is known about the actual use of different functionalities and the effective engagement.

**Objective:**

The aim of this study was to explore the use and the effective engagement of adolescents (aged 12 to 18 years) with the Adolescent Adherence Patient Tool (ADAPT).

**Methods:**

The ADAPT intervention consisted of an app for patients, which was connected to a management system for their pharmacist. The aim of the ADAPT intervention was to improve medication adherence and, therefore, the app contained multiple functionalities: questionnaires to monitor symptoms and adherence, medication reminders, short movies, pharmacist chat, and peer chat. For this study, data of the ADAPT study and a cluster randomized controlled trial were used. Adolescents with asthma had 6 months’ access to the ADAPT intervention, and all app usage was securely registered in a log file.

**Results:**

In total, 86 adolescents (mean age 15.0, SD 2.0 years) used the ADAPT app 17 times (range 1-113) per person. Females used the app more often than males (*P*=.01) and for a longer period of time (*P*=.03). On average, 3 different functionalities were used, and 13% of the adolescents used all functionalities of the app. The questionnaires to monitor symptoms and adherence were used by most adolescents. The total app use did not affect adherence; however, activity in the pharmacist chat positively affected medication adherence (*P*=.03), in particular, if patients sent messages to their pharmacist (*P*=.01).

**Conclusions:**

mHealth apps for adolescents with asthma should contain different functionalities to serve the diverging needs and preferences of individual patients. Suggested key functionalities to promote use and effectiveness in adolescents with asthma are questionnaires to monitor symptoms and a health care provider chat.

## Introduction

Mobile health (mHealth) interventions have the potential to support patients with their medication use and are therefore increasingly used [1-4]. Patients highly appreciate those type of interventions, mainly because of the high usability, feasibility, and acceptability of mHealth [5]. However, the evidence for efficacy of mHealth for chronic patients is limited, except for moderate quality evidence of improvement in asthma patients [3].

mHealth seems, in particular, promising for specific patient groups such as adolescents because almost all adolescents own a smartphone (95%); they widely use their phone for social networking, and they are generally poor adherents [6,7]. However, until now, not many mHealth interventions are developed for adolescents, although mHealth interventions for adolescents were rated as feasible and acceptable with modest evidence for their efficacy in improving adherence [8-10]. Therefore, we developed the Adolescent Adherence Patient Tool (ADAPT), an interactive mHealth intervention to improve medication adherence in adolescents with asthma. A patient-centered approach and a theoretical framework were used to develop this intervention [11]. As a result, the intervention consisted of a smartphone app for patients, which was connected to a desktop application for pharmacists, enabling communication between patients and health care providers.

Previous studies showed that multifaceted mHealth interventions are more effective in improving medication adherence than interventions targeting only 1 aspect of nonadherent behavior [4,12-14] because medication adherence is a complex behavior affected by many factors [15]. Accordingly, the ADAPT intervention contained multiple functionalities to support medication adherence: questionnaires to monitor symptoms and adherence, medication reminders, short movies, pharmacist chat, and peer chat [11]. We evaluated the ADAPT intervention in a cluster randomized controlled trial and adherence improved significantly in adolescents with asthma having poor adherence rates [16].

Besides the efficacy of mHealth, it is important to study the actual use of mHealth interventions. Currently, little is known about the actual use of mHealth apps by adolescents with asthma. Moreover, it is important to identify the association between the use of different mHealth functionalities and the effect on the intended outcome, also known as *effective engagement*. This will provide directions for other mHealth interventions aiming to improve adherence, as there is still limited evidence for the efficacy of mHealth [17,18]. Therefore, the aim of this study was to explore the use of the ADAPT app, a complex adherence mHealth intervention, by an adolescent with asthma and to study the effective engagement of patients with the ADAPT app.

## Methods

### Data Collection

Data of the ADAPT study, a cluster randomized controlled trial, were used. The aim of the ADAPT study was to evaluate the effect of the ADAPT intervention on adherence, measured with the Medication Adherence Report Scale (MARS) [19]. The complete ADAPT study protocol and effectiveness of the mHealth intervention have been described elsewhere [11,16]. Briefly, adolescents with asthma (aged 12 to 18 years) who were in the possession of a smartphone were eligible for participation. In total, 638 patients were invited for the intervention group and 103 (16.1%) signed the informed consent. There was a 16% dropout rate (n=8 withdrew consent, n=7 did not download the app, and n=1 was lost to follow-up), resulting in 87 patients and 27 pharmacists, who had 6 months access to the ADAPT intervention. The control group consisted of 147 patients and 27 pharmacists (data not shown).

We asked patients in the intervention group (N=87) to use the app at least once a week; they received a weekly push notification. After 6 months upon completing the study, patients received a gift card (regardless of their app usage). All ADAPT app use was securely registered in a log file, that is, a document with an automatically produced and timestamped documentation of events.

### Adolescent Adherence Patient Tool Intervention

**Figure 1:**
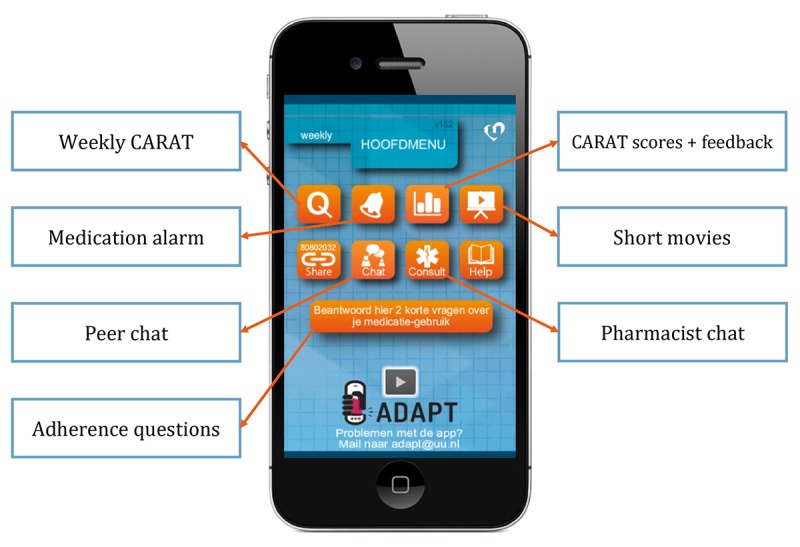
The Adolescent Adherence Patient Tool with the different functionalities. CARAT: Control of Allergic Rhinitis and Asthma Test.

The ADAPT app (Figure 1) was connected to a desktop application of the patient’s own community pharmacist [11]. The different functionalities of the app are described below.

#### Questionnaire to Monitor Symptoms

Patients received a weekly push notification (26 times in total) to complete the Control of Allergic Rhinitis and Asthma Test (CARAT) to monitor their symptoms [20]. This validated questionnaire consisted of 10 questions where a total score between 0 and 30 could be obtained (>24 indicated good disease control). The total score could be divided into 2 subscores: allergic rhinitis score (items 1 to 4, score>8 indicated good control) and asthma score (items 5 to 10, score ≥16 indicated good control). Patients had access to their obtained CARAT scores in the ADAPT app and received textual feedback about their results. The CARAT scores were also sent to the pharmacist’s desktop application, and pharmacists received email notifications when patients had no disease control (CARAT score ≤24).

#### Medication Alarm

Patients could set a medication alarm to prevent forgetting. The alarm was adjustable to the patient’s preferences, that is, patients could set the alarm once or twice a day at their preferred time. The alarm was not connected to their inhaler medication; thus, it did not register if medication was already taken. Unfortunately, use of the medication alarm was not registered in the log file, as the alarm settings were saved locally.

#### Short Movies

Almost every week a short movie about an asthma-related topic (eg, lifestyle, medication use, and friends) became visible in the app to educate and motivate the patient. Patients did not receive a push notification, although in the app a notification was visible when a new movie became available. In total, 21 movies became available during the 6-month study period. Pharmacists had access to the movie database and could send additional movies based on the patient’s needs, for example, about inhaler techniques.

#### Peer Chat

The peer chat gave patients the opportunity to share experiences and discuss asthma-related topics with other participants. This was an age-specific functionality, as peers are important during adolescence [21]. Adolescents recommended this functionality during the developmental phase. The messages were divided over 6 topics: asthma, general, going out, pets, sports, and other. There was no moderator involved as we did not want to disrupt the interaction between adolescent peers.

#### Pharmacist Chat

The pharmacist chat facilitated direct contact between the adolescent and their pharmacist, which is important because adolescents are not often seen in the pharmacy [22]. Pharmacists voluntary signed up for the ADAPT study and were randomized to the intervention group. Pharmacists could contact their *own* patients via the intervention, as in the Netherlands every patient is registered at 1 pharmacy and mostly fill all their prescriptions there. Pharmacists received email notifications when patients sent a message. The aim of this functionality was to educate and motivate patients.

#### Adherence Questions

Once every 2 weeks (14 times in total), 2 questions concerning adherence appeared in the app. The questions were based on items of the MARS. The first question was related to unintentional nonadherence: *How often did you forget to take your medication in the previous week?* and the other was related to intentional nonadherence: *How often did you decided to miss out a dose in the previous week?* Patients could answer these questions using a 5-point Likert scale ranging from 1 (always) to 5 (never).

### Data Analysis

Descriptive statistics of all variables were calculated. For skewed data, the median with interquartile range (IQR) is shown instead of the mean with standard deviation (SD). We divided the adolescents in 3 groups based on the frequency of the app usage during the 6-month study period: low (≤10), average (>10 and ≤25), and high (>25) frequent users. All log file data were converted to Excel and, thereafter, statistical analyses were performed using R (R Foundation for Statistical Computing, version 3.4.3) packages *nlme* and *lme4*. *P* values less than .05 were considered statistically significant.

#### Effective Engagement

We used (generalized) linear mixed-effects models to evaluate the effective engagement of adolescents and to compare groups. The 27 pharmacies of the ADAPT study (clusters) were used as random effects in the models.

### Ethics and Confidentiality

The ADAPT study was approved by the Medical Review Ethics Committee of the University Medical Centre Utrecht (NL50997.041.14) and by the Institutional Review Board of Utrecht Pharmacy Practice network for Education and Research, Department of Pharmaceutical Sciences, Utrecht University [23]. All participants had to sign informed consent before the start of the study; for patients younger than 16 years, both parents also had to sign. The trial is registered in the Dutch Trial Register (NTR5061). All (personal) app data were encrypted using 128-bit Advanced Encryption Standard and were securely saved using Hypertext Transfer Protocol with a Secure Sockets Layer certificate (HTTPS).

## Results

### Descriptives

In total, 87 adolescents (mean age 15.0 SD 2.0 years; 55% females) downloaded the ADAPT app on their smartphone (Table 1), of which 86 adolescents used it 1975 times between October 2015 and April 2017. The median app use per person was 17 times (IQR 6-31; range 1-113) within a period of 5 months (IQR 3-6; range 0-8). Females used the app more often than males (median 20.5 vs 11; *P*=.01) and over a longer period (median 5 vs 6 months; *P*=.03).

The exact use per functionality is described in Table 1; the CARAT questionnaire, adherence questions, and short movies were used by most adolescents. There were differences in characteristics and functionalities used between the 3 user groups: low, average, and frequent users (Table 1). The low frequency app users had lower self-reported adherence rates compared with the average group (MARS 19.3 vs 21.4; *P*=.04), and the high frequency group contained more females compared with the low frequency group (73% (19/26) vs 44% (12/27); *P*=.04). Almost all low frequent users (93%; 25/27) completed the CARAT questionnaire, and more than half (56%; 15/27) completed the adherence questions at least once. No one sent a message in the peer chat. The majority of high frequent users sent a message to their pharmacist (81%; 21/26) and watched a movie (77%; 20/26), which differed significantly from the other groups (Table 1).

**Figure 2:**
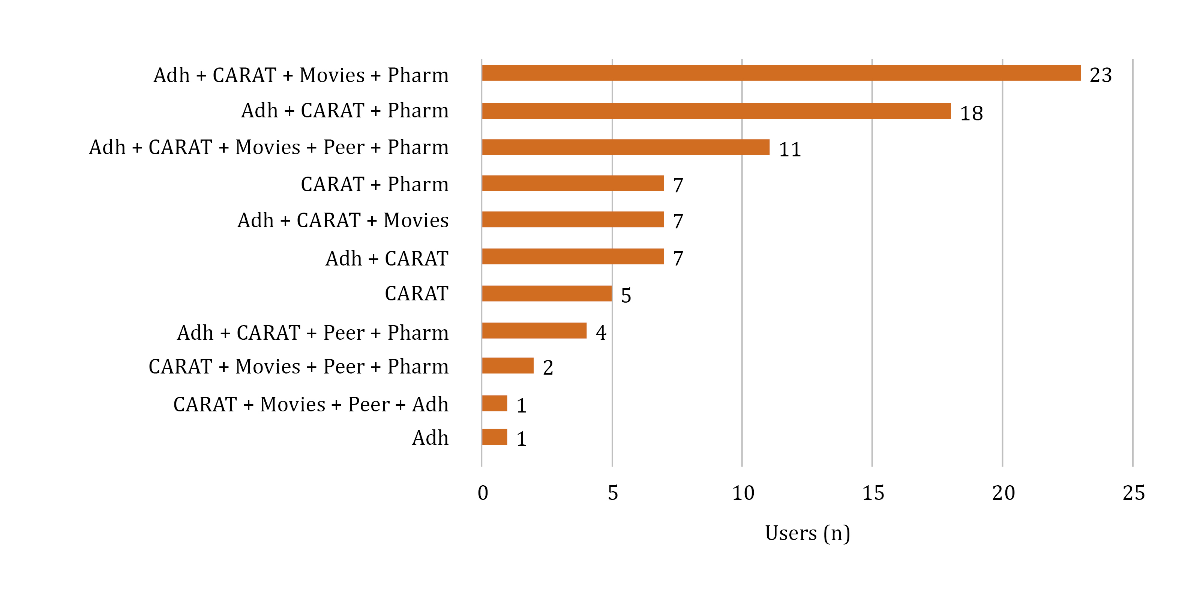
Overview of the combinations of functionalities used by 86 adolescents. Adh: adherence questions; CARAT: Control of Allergic Rhinitis and Asthma Test; peer: peer chat; pharm: pharmacist chat.

Adolescents used, on average, 3 different functionalities of the app (IQR 3-4; range 1-5). An overview of the combinations of different functionalities used is presented in Figure 2, showing a wide variety in app functionality use. All 5 functionalities were used by 13% (11/87) of the adolescents. Examples of the total app usage per person are shown in Multimedia Appendix 1.

### Questionnaire to Monitor Symptoms

The CARAT questionnaire is the most frequently used functionality of the app; in total, 1047 questionnaires were completed by 85 (98%) adolescents (Multimedia Appendix 2). Adolescents received 26 weekly reminders during the study period (6 months) to complete the CARAT; however, they individually completed the CARAT on average 10 times (IQR 4-17). There was a lot of variation between patients; range 1-84.

### Adherence Questions

The majority of adolescents (83%; 72/87) completed the adherence questions at least once, with a total of 221 completed questionnaires. The median of completed adherence questions per person was 2 (IQR 1-4; range 1-11), whereas the adherence questions appeared 14 times during the study period.

### Short Movies

Half of the adolescents (51%; 44/87) watched at least one movie. More females (n=29) than males (n=15) watched movies (*P*=.04). In total, 21 short movies appeared in the app; however, on average, 4 different movies were watched per person (IQR 2-6; range 1-20), and each movie was seen once (IQR 1-1; range 1-4). The movies that appeared first in the app were seen most. In addition, 1 pharmacist sent an additional movie with inhaler instructions to support a patient; this movie was seen twice.

**Table 1:** Descriptives of the adolescent app users and the differences between the frequency groups.

Variable name		Total (N=87)	Low^a^ (n=27)	Average^a^ (n=34)	High^a^ (n=26)	*P* value^b^		
						Low versus average	Low versus high	High versus Average
Female, n (%)		48 (55)	12 (44)	17 (50)	19 (73)	.59	.04	.08
Age (years), mean (SD)		15.0 (2.0)	15.4 (2.0)	15 (2.0)	14.6 (2.1)	.50	.16	.40
Adherence (MARS^c^), mean (SD)		20.4 (3.9)	19.3 (3.9)	21.4 (3.5)	20.3 (4.2)	.04	.30	.35
**Asthma control (CARAT^d^), mean (SD)**		19.3 (5.3)	18.7 (6.0)	19.6 (5.4)	19.5 (4.6)	.52	.72	.80
	Allergic rhinitis	7.3 (3.2)	7.0 (3.5)	7.6 (3.1)	7.0 (3.2)	.48	.98	.47
	Asthma	12.1 (3.2)	11.7 (3.6)	12.1 (3.1)	12.4 (2.8)	.68	.47	.72
Duration app use (months), mean (SD)		4.2 (2.1)	3.0 (2.4)	4.7 (1.6)	6.1 (1.3)	<.001	<.001	.008
**Frequency app use^e^, mean (SD)**		22.5 (22.0)	4.3 (2.6)	17.1 (4.6)	48.6 (22.9)	<.001	<.001	<.001
	CARAT, n (%)	85 (98)	25 (93)	34 (100)	26 (100)	—^f^	—	—
	Adherence, n (%)	72 (83)	15 (56)	31 (91)	26 (100)	.003	—	—
	Short movies, n (%)	44 (51)	7 (26)	17 (50)	20 (77)	.06	<.001	.04
	Pharmacist chat, n (%)	38 (44)	2 (7)	15 (44)	21 (81)	.006	<.001	.009
	Peer chat, n (%)	18 (21)	0 (0)	7 (3)	11 (42)	—	—	.04

^a^Frequency of app use: low=used the app ≤10 times; average=used the app >10 and ≤25 times; high=used the app >25 times.

^b^*P* values derived of (generalized) linear mixed-effects models.

^c^MARS: Medication Adherence Report Scale.

^d^CARAT: Control of Allergic Rhinitis and Asthma Test.

^e^Used at least once.

^f^Not applicable.

**Table 2:** Descriptives of pharmacists using the pharmacist chat.

Pharmacists who started the chat (n=53)	n (%)	No response, n (%)
Question about the CARAT^a^ score	24 (45)	13 (25)
Welcome message	16 (30)	10 (19)
Welcome message and question	7 (13)	2 (4)
Comment on the CARAT score	4 (8)	1 (2)
Question	2 (4)	1 (2)

^a^CARAT: Control of Allergic Rhinitis and Asthma Test.

**Table 3:** Descriptives of patients using the pharmacist chat.

Patients who started the chat (n=12)	n (%)	No response, n (%)
Question about medication	5 (42)	3 (25)
Question about the app, asthma, or general	4 (33)	0
General comment	2 (17)	1 (8)
Medication comment	1 (8)	0

### Pharmacist Chat

In total, 65 of the 87 adolescents (75%) sent or received 3 chat messages within the pharmacist chat (IQR 1-7; range 1-37), with a total of 347 messages. In most cases (82%; 53/65), the pharmacist started the chat; however, half of those pharmacists (51%; 27/53) did not receive a chat message back (Table 2). In general, the majority of pharmacists (82%; 22/27) sent messages with a median of 2 messages per adolescent (IQR 1-5; range 1-20).

Of the 12 adolescents who started the conversation, one-third (n=4) did not receive a message back (Table 3; reasons unknown). In total, 38 adolescents (44%) sent on average 2 messages (IQR 1-5; range 1-17) to their pharmacist, and more females (n=28) than males (n=10) sent messages to their pharmacist (*P*=.004). In total, 34 conversations were held where both pharmacists and patients sent at least 1 message; examples are shown in Multimedia Appendix 3.

### Peer Chat

The peer chat was used by 21% (18/87) of the adolescents; in total, they sent 150 chat messages. Per adolescent, 4.5 messages (IQR 3-11; range 2-29) were sent. Most messages were sent within the topics *sports* (67 messages by 8 adolescents), *other* (34 messages about age, school, and residence by 6 adolescents), and *general* (24 messages about participating in the study and the app by 8 adolescents). The 18 adolescents participated on average in 2 topics (IQR 1-2; range 1-5). Examples of peer chat messages are shown in Multimedia Appendix 3.

### Effective Engagement

The total app use was not associated with a difference in self-reported adherence (*P*=.12). Use of the CARAT questionnaire (*P*=.26), adherence questions (*P*=.65), short movies (*P*=.80), or peer chat (*P*=.21) also did not affect the adherence outcome. However, logged activity in pharmacist chat positively affected self-reported adherence (MARS score increased with 0.1 points per message; *P*=.03). Data showed that messages sent by pharmacists were not related to the outcome (*P*=.06), whereas activity of patients in the pharmacist chat did positively affect the outcome (*P*=.01), that is, if patients sent messages to their pharmacist, it positively affected adherence (MARS score increased with 0.3 points per chat message).

## Discussion

### Principal Findings

Adolescents have different preferences when using an mHealth app, as there was a wide variety in app usage per person. This supports the need for multifaceted mHealth interventions. The questionnaire to monitor symptoms was the frequently used functionality, for which they received weekly reminders. Females seemed to be more active in the ADAPT app; they used the app more often, for a longer duration, and more females sent messages to their pharmacists and watched movies. Total app use was not associated with the outcome; however, sending a chat message to the pharmacist positively affected medication adherence. On the basis of our results, we recommend a health care provider chat as a key functionality for mHealth interventions to improve adherence in adolescents with asthma.

The ADAPT intervention contained a unique combination of functionalities to improve adherence and targeted a specific patient population: adolescents with asthma. We showed that the adolescents who used the app for 10 to 25 times (average users) had the highest adherence score at the start (MARS 21.4). One would expect the highest adherence score among the low frequent users because if patients are highly adherent, they do not need the intervention, or that among the high frequent users, as they are also likely to be highly adherent to the intervention use. However, we did not find this, although there was no difference between adherence rates among average and high users; thus, higher adherence rates might be related to more frequent app use, that is, more adherent to the intervention.

The most used functionality was the questionnaire to monitor symptoms (Table 1), which was also shown in a study with adult asthma patients [24]. The symptom questionnaire provides patients (and their health care providers) insights into their disease symptoms over time, which should support self-management [25,26]. Surprisingly, we did not find an effect of the questionnaire use on adherence. Patients received a weekly push notification to complete the questionnaire, which might explain why this functionality was the most used functionality. However, the adherence questionnaire was the second most used functionality (Table 1), for which patients did not receive a push notification. The reason why most patients completed the questionnaires is unknown, among others, curiosity might play a role. On the basis of all these questionnaire data (adherence and symptom control), health care providers could deliver personalized care to support patients, which is suggested to be more effective than usual care [27-30]. Therefore, we recommend questionnaires as a useful functionality for mHealth aimed at adolescents.

The peer chat was an age-specific functionality based on the preferences of adolescents [11,31] because peers are important for them [32]. Previous studies showed positive effects of peer-led interventions for asthma patients in improving attitudes and quality of life [33,34], and online peer support groups increased self-confidence [35]. In our study, no effects of the peer chat were found on adherence. Only 21% of the adolescents (18/87) used the peer chat, suggesting that it was not appropriate for everyone. However, the adolescents who used it sent quite a lot of messages (8 per person). Therefore, more research is needed toward a peer chat functionality in a larger population, as more interaction is expected when more patients participate, which in turn might support the use of the peer chat.

The pharmacist chat is a new communication method for both patients and pharmacists. It provided pharmacists with a tool to personally reach patients, which is in particular relevant for adolescent patients, as their adherence is low and they are not often seen in the pharmacy [22]. This electronic consult might overcome patient’s barriers to approach a health care provider. However, this study showed that not all adolescents and pharmacists were comfortable with using this new tool because only 44% of the adolescents (38/87) and 82% of the pharmacists (22/27) used the ADAPT pharmacist chat. Moreover, 4 adolescents (with different pharmacists) did not receive an answer to their question or comment (Table 3). For further implementation of mHealth, it is important that patients always receive an answer, otherwise it will hinder further implementation [36]. Health care providers should therefore be stimulated and motivated to actively engage in mHealth, and we suggest a back-up plan, for example, automatically sending personalized short message service text messages for patients who did not receive an answer within 24 hours or an urgent email notification for pharmacists.

For further implementation of mHealth in clinical practice, it is important to study the cost-effectiveness of the ADAPT intervention. Most mHealth interventions are cost-effective [37]; however, the active involvement of health care providers, in our case pharmacists, might negatively affect the cost-effectiveness. Thus, comprehensive economic evaluations are needed [38] to study the cost-effectiveness of the ADAPT intervention and to identify the optimal involvement of pharmacists (from an economical perspective).

### Limitations

We used log data to analyze the ADAPT app usage, which is a reliable method; however, there are some limitations. Data used in this study are derived from a cluster randomized controlled trial; thus, there might be a response bias, that is, the participants were probably more motivated to use the intervention than the general population. However, use of the intervention still varied per person, suggesting that mHealth use depends on patients’ needs and preferences. Another limitation is that patients received a weekly reminder to complete the CARAT questionnaire, which might be a reason why the CARAT is mostly used. Moreover, many researchers are using electronic monitors to measure adherence of youth with asthma; thus, further research should focus on effective engagement using electronic adherence measurements instead of self-reports. In addition, we studied the physical engagement of adolescents with the app (number of times used), although there is also psychological engagement with the intervention [17,39], which we did not measure. The psychological engagement might also explain why patients use certain functionalities. Moreover, the generalizability of our results is limited because our findings are based on a study among adolescents with asthma in the Netherlands. Therefore, more research is needed to confirm our findings in other countries and populations. However, these results suggest that the possibility to chat with a health care provider is an important functionality for mHealth interventions aiming to increase adherence.

### Conclusions

This study showed that a complex mHealth intervention to support adherence is used differently by adolescents with asthma. The questionnaires to monitor asthma symptoms and adherence were used by most adolescents, which provided valuable data for health care providers and patients. Moreover, the use of the pharmacist chat positively affected adherence. These findings suggest that mHealth apps should contain different functionalities to serve the diverging needs and preferences of individual patients. A questionnaire to monitor symptoms and adherence and a chat with the health care provider are recommended key functionalities for mHealth apps for adolescents with asthma.

## Appendix

Multimedia Appendix 1 Examples of mobile health (mHealth) app use by adolescents with asthma during the 6-month study period, divided in low (1a), average (1b), and high frequent (1c) users. 1a. Low frequent users who used the intervention 4 (left) and 5 times (middle and right). 1b. Average frequent users who used the intervention 17 (left), 18 (middle), and 22 times (right). 1c. High frequent users who used the intervention 40 (left), 43 (middle), and 50 times (right). Horizontal lines: Red: Control of Allergic Rhinitis and Asthma Test (CARAT) score, dotted line represents the threshold (>24); Blue: adherence questions; Light blue: forgot to take, Dark blue: decided not to take (5=never, 1=always). Vertical lines: Green: pharmacist chat; Dark green: message sent by pharmacist; Light green: message sent by patient; Pink: watched a movie; Black: dotted line indicates the baseline and end of follow-up date of the study; Grey: dotted line indicates the push notification update of the mHealth app.

Multimedia Appendix 2 Overview of the 1047 Control of Allergic Rhinitis and Asthma Test (CARAT) scores of 85 adolescents; on average 12.3 scores per person. Blue: Total CARAT score, dotted line represents the threshold (>24); Green: Asthma subscore, dotted line represents the threshold (≥16); Red: Allergic rhinitis subscore, dotted line represents the threshold (>8).

Multimedia Appendix 3 Examples of chat messages sent in the Adolescent Adherence Patient Tool.

## Abbreviations

ADAPT: Adolescent Adherence Patient Tool
CARAT: Control of Allergic Rhinitis and Asthma Test
IQR: interquartile range
MARS: Medication Adherence Report Scale
mHealth: mobile health
SD: standard deviation
