# Supplementary figures and images for: Effective Engagement of Adolescent Asthma Patients With Mobile Health–Supporting Medication Adherence
Source: JMIR Mhealth Uhealth. 2019 Mar 27;7(3):e12411. doi: 10.2196/12411 (PMC6456831; doi:10.2196/12411)

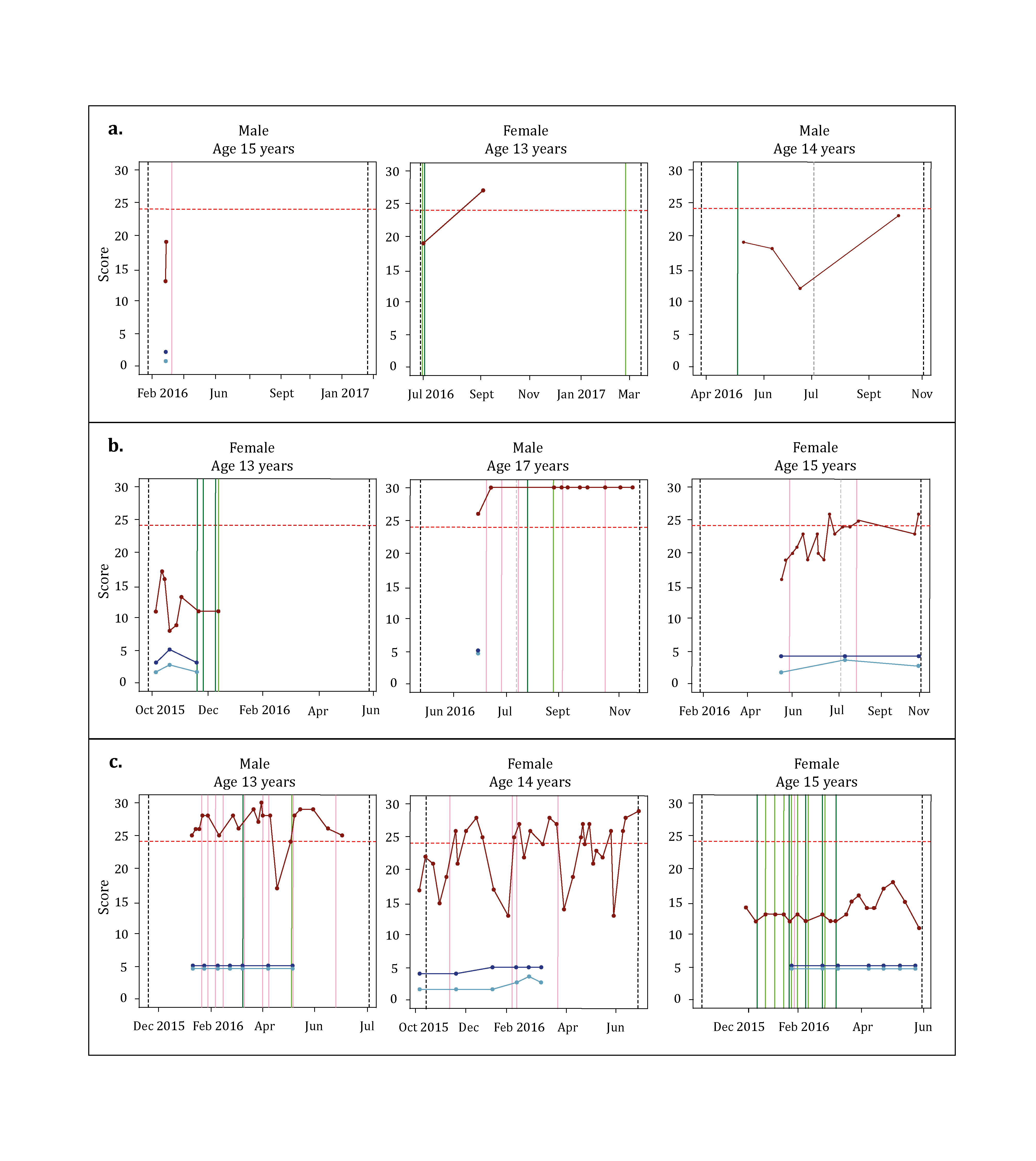

Supplement: Multimedia Appendix 1 [file mhealth_v7i3e12411_app1.png]

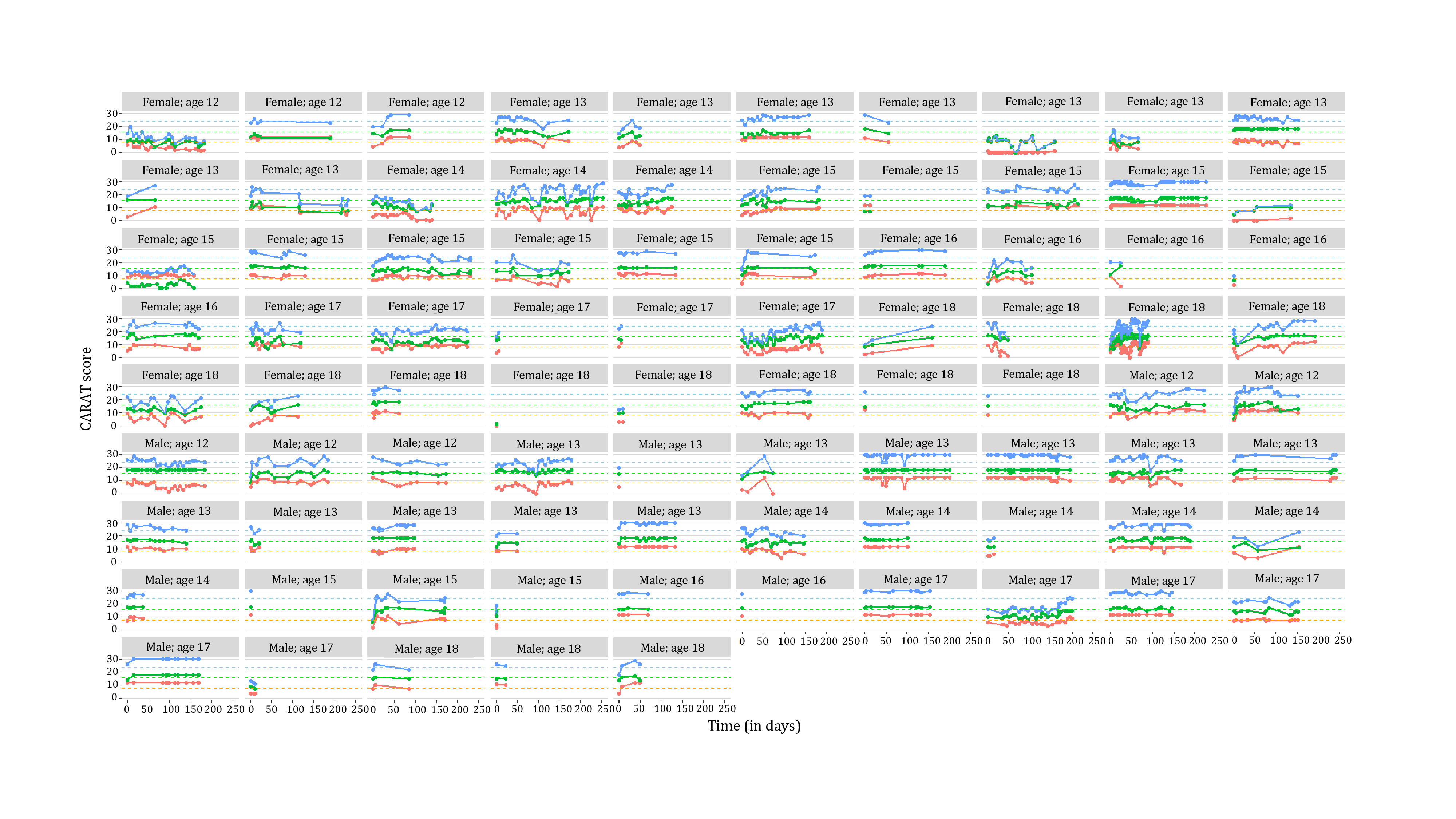

Supplement: Multimedia Appendix 2 [file mhealth_v7i3e12411_app2.png]
