# Supplementary material for: Effective Engagement of Adolescent Asthma Patients With Mobile Health–Supporting Medication Adherence
Source: JMIR Mhealth Uhealth. 2019 Mar 27;7(3):e12411. doi: 10.2196/12411 (PMC6456831; doi:10.2196/12411)
Supplement: Multimedia Appendix 3 [file mhealth_v7i3e12411_app3.docx]

| **Chat (Topic)** | **Sender** | **Message** |
| --- | --- | --- |
| Pharmacist chat | Female;  15 years | “Is it necessary to take the medication at a fixed time?” |
|  | Pharmacist | “If you do not take your medication at a fixed time, you may forget to take it. Your medication will be less effective then.” |
| Pharmacist chat | Pharmacist | “How are you? Your questionnaire scores are quite low. Is there anything I can help you with? Do you want to practice your inhaler techniques in the pharmacy?” |
|  | Female;  18 years | “I still have a cold, but I think my inhaler technique is fine.” |
|  | Pharmacist | “How often do you take your medication?” |
| Peer chat (Sports) | Female;  13 years | "Do you suffer a lot when playing sports?" |
|  | Female;  13 years | "I only suffer during endurance running, but my trainers take that into account. Although I’m not able to run the shuttle run test at school, because it is very stuffy and dusty, and I’m surrounded by many people." |
| Peer chat (Other) | Female;  18 years | "Does anyone have experience with hay fever tape? Can a physiotherapist do this?" |
